# Proton pump inhibitors and acute kidney injury: a nested case–control study

**DOI:** 10.1186/1471-2369-14-150

**Published:** 2013-07-16

**Authors:** Donald G Klepser, Dean S Collier, Gary L Cochran

**Affiliations:** 1 Department of Pharmacy Practice, College of Pharmacy, University of Nebraska Medical Center, 986045 Nebraska Medical Center, Omaha, NE 68198-6045, USA

**Keywords:** Proton pump inhibitors, Kidney failure, Renal disease, Nephritis

## Abstract

**Background:**

Proton pump inhibitors (PPI) are a widely-used class of drugs for the treatment of gastro-esophageal reflux disease and other acid-related disorders of the gastrointestinal tract. As a class, PPIs have demonstrated a favorable safety profile. However, case reports have suggested that this class of drugs may be linked to acute kidney injury, which may in turn lead to chronic injury or failure. The objective of this study was to determine if an association between PPIs and kidney failure exists and to estimate an effect size for the relationship between PPI use and renal disease.

**Methods:**

A nested case–control study was conducted in a privately insured population in a single Midwestern state including a total of 184,480 patients aged 18 years or older who were continuously enrolled with the insurer for at least 24 months between September 2002 and November 2005.

Of the patients eligible for the study, 854 cases were identified as having at least two claims for an acute renal disease diagnosis. Cases were randomly matched with up to four controls (n = 3,289) based on age, gender, county of residence, and date of entry into the cohort. Patient demographic data, PPI use, illnesses, and medications associated with renal disease and a proxy for health status using pre-existing patient comorbidities were collected from inpatient, professional, and prescription claims data. Conditional logistic regression models were used to evaluate the association between renal disease and PPI use.

**Results:**

Renal disease was positively associated with PPI use (odds ratio [OR] 1.72, 95% confidence interval [CI] 1.27, 2.32, p < 0.001) even after controlling for potential confounding conditions. After removing patients with potential confounding disease states from the study population, the number of cases (195 of the 854) and controls (607) was lower, but the relationship between renal disease and PPI use remained consistent (OR 2.25, CI 1.09-4.62, p < 0.001).

**Conclusions:**

Patients with a renal disease diagnosis were twice as likely to have used a previous prescription for a PPI. Therefore, it is necessary for physicians to increase recognition of patient complaints or clinical manifestations of this potentially harmful event in order to prevent further injury.

## Background

Proton pump inhibitors (PPI) are a widely-used class of drugs for the treatment of gastro-esophageal reflux disease and other acid-related disorders of the gastrointestinal tract. In 2009, more than 119 million PPI prescriptions were filled in the United States, accounting for nearly $14 billion dollars in prescription PPI sales, in addition to billions more in over-the-counter (OTC) sales [1]. The PPI class includes several different agents, such as esomeprazole, omeprazole, lansoprazole, pantoprazole, deslansoprazole, rabeprazole, etc., all of which possess a common mechanism of action for reducing parietal cell acid production by blocking H+/K + adenosine triphosphatase [2,3].

As a class, PPIs have demonstrated a favorable safety profile [4-6]. Overall, complications have been infrequent and minor. The most common unwanted effects reported have been headaches, abdominal pain, and diarrhea [2]. In fact, the favorable adverse effect profile has led to movement of some of these agents to non-prescription status. However, as the use of PPIs has grown more widespread so have safety concerns. A growing body of literature suggests that this class of drugs may be linked to acute kidney injury, which can potentially lead to chronic injury or kidney failure. In 1992, a sentinel case-report identified the PPI omeprazole as a possible cause of acute interstitial nephritis (AIN) in an elderly woman [7]. Since that time, numerous biopsy-confirmed case reports and retrospective descriptive reports have been published suggesting a connection between PPI use and AIN [8-10]. Detailed summaries of these case reports have been published [9,11,12]. However, research in this area has been limited and consisted of small sample sizes without comparator groups. As a result, no estimate of the effect size is currently available. In turn, determining if an association between PPI use and AIN exists requires studies using larger sample sizes and control groups.

Drug-induced AIN is a form of acute kidney injury thought to result from an idiosyncratic, cell-mediated immunologic reaction [13,14]. Drugs are the most commonly cited cause of AIN, but infectious and immune etiologies are also possible [15]. Recognition of AIN is elusive as the clinical manifestations are subtle. Classically, AIN has been described to present the following triad of events: fever, rash, and arthralgias [16-18]. Unfortunately, in case-reports of PPI-induced AIN, the triad of symptoms was present in a minority of subjects. Most complaints were nonspecific, including fever, fatigue/lethargy, weight loss, and nausea/vomiting [9,11,12]. Laboratory abnormalities were more consistently present in PPI-induced AIN cases, with elevated serum creatinine, pyuria, proteinuria, and hematuria observed in over 60% of the cases [9,11,19]. Because of varied clinical presentation and lack of accurate non-invasive tests, AIN can only be definitively diagnosed through a renal biopsy [13,18].

In practice, PPI-induced AIN is likely under- or mis-diagnosed due to low suspicion (infrequent occurrence, limited information dissemination) and inconsistent clinical presentation [12]. Rather, AIN is more likely to be classified as some other form of acute or chronic renal injury. Recognizing this mis-classification, studies using claims data to investigate a link between PPI use and AIN require broadening ICD-9 codes beyond the single heading “AIN”. The objective of this study was to determine if an association exists between renal injury and PPI use and in turn estimate an effect size for this association using claims data.

## Methods

This retrospective nested case–control study used claims data from a private insurer in a single Midwestern state to examine the relationship between renal disease and prescription PPI use. The nested case–control design has been employed in pharmacoepidemiologic studies, and its strengths and limitations have been previously described [20-22]. The insurer’s databases included 39 months (September 2002 through November 2005) of inpatient, outpatient, and prescription claims for a cohort of 184,480 patients. The database has been used in a number of studies and is a generally complete claims record for covered patients [23-25]. Patient identifiers were encrypted but could be linked across medical and prescription claims databases and years as long as the patient had coverage during these years. The study was approved by the University of Nebraska Medical Center Institutional Review Board.

### Study cohort

Using demographic data and dates of coverage provided by the insurer, patients under the age of 18 and with less than two years of continuous coverage during the study period were excluded from the study. Patients diagnosed with renal disease (Table 1) within their first 12 months of entry into the cohort (i.e., the beginning of the study period or start of their continuous coverage) were also excluded from the study. Patients included in the study were 18 years or older, diagnosed with renal disease for at least 12 months, and continuously enrolled with the insurer for at least 24 months between September 2002 and November 2005. These inclusion and exclusion criteria assured that all patients had at least twelve months of claims data prior to the onset of renal disease.

**Table 1:** **ICD-9**^**a **^**codes used to identify cases and pre-existing renal disease**

**Description**	**Pre-existing renal disease**	**Cases**
Hypertensive renal disease	403.XX	
Acute glomerulonephritis	580.XX	580.89
Nephrotic syndrome	581.XX	
Chronic glomerulonephritis	582.XX	
Nephritis and nephropathy	583.XX	583.81, 583.89, 583.9
Acute renal failure	584.XX	584.8, 584.9
Chronic renal failure	585.XX	
Renal failure, unspecified	586.XX	586.XX
Impaired renal function disease NEC^b^	588.89	
Unspecified disorder of kidney and ureter	593.9	593.9
Kidney transplant	V420	
Dialysis	V451, V56X	

^a^*ICD-9* International Classification of Diseases, Ninth Revision.

^b^*NEC* not elsewhere classifiable.

### Case and control definition

Given that the true nature of PPI-induce renal injury (i.e., AIN) is frequently misdiagnosed, multiple ICD-9 codes for acute or chronic renal disease were used to capture un-recognized cases. A practicing nephrologist was asked to identify renal disease diagnoses (Table 1) within the universe of ICD-9 codes that could be used to bill for AIN. Cases were defined as patients with at least two renal disease claims following 12 months of coverage within the study period. At least two claims were required to ensure that the first claim was not merely a diagnostic test used to rule out a renal disease.

Each case was then matched, using incident density sampling based on age, gender, county, and initial date of coverage with up to four randomly selected controls. Cases and controls were matched with the initial date of insurance coverage to account for changes in treatment patterns over the course of the study and to allow for the assignment of a common index date for each case and its controls. The index date was the service date of each case’s first renal disease claim and was assigned to both the cases and matched controls to determine the timing of PPI use and the presence of potential confounders at the time of diagnosis.

### Covariates

PPI exposure status was obtained through prescription claims. Exposure was defined as having a PPI claim in the 90 days prior to the index date. Recognizing that our definition of renal injury includes cases not related to PPI use, we used a multi-variable regression to control for the effect of these confounding causes of renal injury. Potential confounders in this study were illnesses and the use of prescription medications that have been previously shown to be associated with renal disease [26]. These potential cofounders include diabetes, hypertension, high cholesterol, and antibiotic, diuretic, or use of non-steroidal anti-inflammatory drugs (NSAID). Use of OTC medications is not captured in a claims data base. Confounding by health status is an important consideration because patients with a lower health status are more likely to 1) have a renal disease diagnosis and 2) use PPIs [27]. As a proxy for patient health status, we included comorbidities from the Deyo modification of the Charlson comorbidity index as potential cofounding variables that were not previously included (i.e., myocardial infarction, congestive heart failure, peripheral vascular disease, cerebrovascular disease, hemiplegia or paraplegia, dementia, chronic pulmonary disease, rheumatologic disease, mild liver disease, moderate or severe liver disease, AIDS, malignancy, and metastatic solid tumor) [28]. All variables were extracted from inpatient, outpatient, and prescription claims data using appropriate ICD-9 and NDC codes. Claims were not generated for OTC PPI or NSAID purchases and therefore this utilization was not captured. The presence of a confounding variable was concluded if a claim was identified in the period (minimum 12 months) prior to the index date.

### Data analysis

We elected to explore the association between PPI-use and renal injury using two models. The first model attempts to control for potential confounding variables associated with renal disease, as listed above in the Covariates section. Conditional logistic regression models were used to calculate odds ratios (ORs) and 95% Confidence Intervals (CIs). All potential predictors were first evaluated in univariable conditional logistic regression models. Those covariates reaching a univariable significance level of p ≤ 0.1 were included in the final multivariable model. The p values corresponding to regression models are based on the Wald test.

Recognizing that some residual confounding variables could remain in the first model, a second conditional logistic regression model excluded cases and controls with pre-existing diagnoses for the comorbidities included in the primary model. As an additional sensitivity analysis, a conditional logistic regression model was used that included propensity scores. The propensity score and predicted probabilities of using a PPI were computed using all remaining independent variables. Finally, the authors conducted an analyses using Histamine-2 Receptor Antagonists (H2 Blockers), which have not been linked to acute kidney injury. All analyses were generated using SAS/STAT software, Version 9.2 of the SAS System for Windows (Copyright SAS Institute Inc.). SAS and all other SAS Institute Inc. product or service names are registered trademarks or trademarks of SAS Institute Inc., Cary, NC, USA.

## Results

In the primary model, 854 cases of renal disease were matched to 3,289 controls. Of the 854 cases, 727 (85.1%) were matched to four controls, while the remaining 127 (14.9%) were matched to three controls. As Table 2 shows, most of the identified renal disease diagnoses were for acute renal insufficiency and acute renal failure. Cases were more likely to use a PPI and were considerably sicker, higher prevalence chronic diseases, (Table 3) than controls. Renal disease was positively associated with PPI use (OR = 2.04; CI 1.53, 2.71) even after controlling for potential confounding conditions (Table 4). Given the incidence in this population (0.0046), the number needed to harm equals 303 patients.

**Table 2:** Cases by diagnosis frequency

**ICD-9**^**a**^	**Description**	**Primary model (n = 854)**	**Secondary model (n = 195)**
580.89	Acute interstitial nephritis	3 (0.35%)	0 (0.00%)
583.81	Nephritis NOS in other disease	17 (1.99%)	1 (0.50%)
583.89	Nephritis NEC^b^	4 (0.47%)	1 (0.50%)
583.9	Nephritis NOS^c^	31 (3.63%)	8 (4.00%)
584.8	Acute renal failure NEC	1 (0.12%)	0 (0.00%)
584.9	Acute renal failure NOS	147 (17.21%)	30 (15.00%)
586	Renal failure NOS	47 (5.50%)	6 (3.00%)
593.9	Acute renal insufficiency	605 (70.84%)	149 (77.00%)

^a^*ICD-9* International Classification of Diseases, Ninth Revision.

^b^*NEC* not elsewhere classifiable.

^c^*NOS* not otherwise specified.

**Table 3:** Characteristics of cases and controls

**Variable**	**Cases (n = 854)**	**Controls (n = 3289)**	**OR**^**a**^	**95% CI**^**b**^	**P value**
Age^c^	51.09 (9.53)	51.10 (9.40)			
Gender^c^					
Female	397 (46.5%)	1525 (46.4%)			
Male	457 (53.5%)	1764 (53.6%)			
PPI^d^ use	126 (14.8%)	191 (5.8%)	2.91	(2.28-3.72)	< 0.001
NSAID^e^ use	293 (34.3%)	703 (21.4%)	1.97	(1.67-2.33)	< 0.001
Antibiotic use	202 (26.7%)	223 (6.8%)	4.41	(3.55-5.47)	<0.001
Diuretic use	215 (25.2%)	293 (8.9%)	3.50	(2.86-4.27)	<0.001
Diabetes	175 (20.5%)	216 (6.6%)	3.94	(3.14-4.94)	< 0.001
Hypertension	302 (35.4%)	626 (19.0%)	2.48	(2.08-2.95)	< 0.001
High cholesterol	238 (27.8%)	659 (20.0%)	1.58	(1.32-1.89)	< 0.001
Myocardial infarction	13 (1.5%)	18 (0.5%)	2.86	(1.40-5.84)	0.004
Congestive heart failure	38 (4.5%)	8 (0.2%)	18.20	(8.48-39.06)	< 0.001
Cerebrovascular disease	15 (1.8%)	12 (0.4%)	5.28	(2.42-11.52)	< 0.001
Peripheral vascular disease	26 (3.0%)	17 (0.5%)	6.19	(3.31-11.56)	< 0.001
Paralysis	6 (0.7%)	4 (0.1%)	5.99	(1.69-21.24)	0.006
Chronic lung disease	45 (5.3%)	47 (1.4%)	3.94	(2.58-6.00)	< 0.001
Liver disease	16 (1.9%)	9 (0.3%)	6.94	(3.06-15.72)	< 0.001
Malignancy	49 (5.7%)	28 (0.9%)	6.90	(4.33-10.97)	< 0.001
Metastatic cancer	27 (3.2%)	9 (0.3%)	12.00	(5.64-25.51)	< 0.001
Rheumatoid arthritis	13 (1.5%)	10 (0.3%)	4.95	(2.16-11.35)	< 0.001
Osteoarthritis	72 (8.4%)	184 (5.6%)	1.57	(1.18-2.10)	0.002

^a^*OR* odds ratio.

^b^*CI* confidence interval.

^c^ Cases and controls were matched on age and gender so those variables were not compared statistically.

^d^*PPI* proton pump inhibitor.

^e^*NSAID* nonsteroidal anti-inflammatory drugs.

**Table 4:** Odds ratios of renal disease from a multiple regression model

**Variable**	**Adjusted OR**^**a**^	**95% CI**^**b**^	**P value**
PPI^c^ use	1.72	(1.27-2.32)	< 0.001
NSAID^d^ use	1.64	(1.35-2.00)	< 0.001
Antibiotic use	3.66	(2.86-4.68)	<0.001
Diuretic use	2.28	(1.78-2.92)	<0.001
Diabetes	2.74	(2.09-3.60)	< 0.001
Hypertension	1.51	(1.21-2.89)	< 0.001
High cholesterol	1.10	(0.89-1.37)	0.388
Myocardial infarction	0.63	(0.21-1.89)	0.409
Congestive heart failure	9.51	(3.90-23.20)	< 0.001
Cerebrovascular disease	3.65	(1.28-10.43)	0.016
Peripheral vascular	4.20	(1.83-9.66)	< 0.001
Paralysis	1.37	(0.25-7.34)	0.717
Chronic lung disease	1.81	(1.03-3.17)	0.040
Liver disease	7.38	(2.88-18.91)	< 0.001
Malignancy	3.72	(2.07-6.70)	< 0.001
Metastatic cancer	8.86	(3.35-23.44)	< 0.001
Rheumatoid arthritis	2.62	(0.97-7.13)	0.058
Osteoarthritis	0.83	(0.60-1.21)	0.373

^a^*OR* odds ratio.

^b^*CI* confidence interval.

^c^*PPI* proton pump inhibitor.

^d^*NSAID* nonsteroidal anti-inflammatory drugs.

After removing patients with potential confounding disease states from the study population, the number of cases (195) and controls (607) decreased (Table 5). Despite the smaller population, the relationship between renal disease and PPI use remained consistent (OR = 2.25; CI 1.09, 4.62).

**Table 5:** Seondary analysis

**Variable**	**Cases**	**Controls**	**Adjusted OR**^**a**^	**95% CI**^**b**^	**P value**
Age (SD)^c^	45.49 (11.39)	44.31 (11.18)			
Gender^c^					
Female	109 (55.9%)	351 (57.8%)			
Male	86 (44.1%)	256 (42.2%)			
PPI^d^ use	15 (7.7%)	23 (3.8%)	2.25	(1.09-4.62)	0.027
NSAID^e^ use	58 (29.7%)	125 (20.6%)	1.36	(0.91-2.03)	0.131
Antibiotic use	45 (23.1%)	48 (7.9%)	3.24	(2.06-5.10)	<0.001
Diuretic use	12 (6.2%)	8 (1.3%)	5.08	(1.95-13.21)	<0.001

^a^*OR* odds ratio.

^b^*CI* confidence interval.

^c^ Cases and controls were matched on age and gender so those variables were not compared statistically.

^d^*PPI* proton pump inhibitor.

^e^*NSAID* nonsteroidal anti-inflammatory drugs.

The inclusion of a propensity score in the model did not change the relationship between PPI use and renal disease (OR 2.05, CI 1.52, 2.72). Analyses of H2 Blockers showed that there was not a statistically significant relationship between H2 Blocker use and renal disease (OR 1.37, CI 0.46, 4.13).

To account for the potential that a 90 day exposure status may include patients not currently using PPIs, sensitivity analyses using a 15 and 30 exposure window were also conducted. The results, not presented, were consistent with and confirmed the base case analyses. Similarly, inclusion of just incident users (first PPI prescription within 90 days) confirmed the results of the base case analyses.

## Discussion

Previous publications have suggested an association between the use of PPIs and acute interstitial nephritis [7-12]. Our study is the first to use a quasi-experimental design to support an association between renal disease and PPI exposure even after controlling for other known causes of renal disease. Additionally, our study provides the first estimate of an effect size for this relationship. We found that patients with an incident of renal disease diagnosis were nearly *twice* as likely to have been exposed to PPIs compared to those without renal disease.

Presently, AIN is not preventable due to its idiosyncratic nature. Therefore it is important that emphasis be placed on timely recognition. Early detection and treatment (i.e., removal of the offending agent and possible use of oral corticosteroids) have been shown to reduce the morbidity of AIN [29]. Healthcare professionals are recommended to have a heightened awareness of patient complaints or clinical manifestations associated with AIN and an understanding of their possible association with PPIs. Pharmacists in particular are in a strategic position to link changes in a patient’s health status with recent PPI utilization.

Like all case–control studies, this analysis has limitations. Misclassification bias can occur if subjects are inaccurately classified regarding the outcome of interest (i.e., renal disease) or the exposure being investigated (i.e., PPI use). By more broadly defining PPI-associated renal disease, we were able to capture more cases, but we were also likely to have included instances of renal injury not associated with PPI exposure. This misclassification of cases could overestimate or underestimate the true relationship between PPI use and renal disease, depending on their distribution between exposure categories. Because our primary and secondary analysis controlled for or removed potential confounders from the analysis, we believe that the misclassified cases are not likely to be associated with PPI use, which is expected to make our estimate conservative.

Lack of OTC PPI utilization can lead to misclassification of exposure. Because OTC use was not captured in a claims database, it is possible that some subjects who used OTC PPIs were misclassified as non-users. Table 3 shows that cases, patients with a renal disease diagnosis, were more likely to be prescribed PPIs than controls. It is likely, given the differences in underlying comorbidities, that cases also used more OTC PPIs. This misclassification of cases would underestimate the effect of PPI injury and make a positive finding more difficult, leading to a more conservative estimate of association.

Surveillance bias could overestimate the impact of PPI exposure. Surveillance bias can occur, as Gordis described, “[i]f a population is monitored over a period of time, disease ascertainment may be better in the monitored population than in the general population…which leads to an erroneous estimate of the relative risk or odds ratio” [30]. In our study, individuals with renal disease were more likely to have an underlying chronic disease (Table 3). If subjects with chronic disease were more likely to see a physician, it is also more likely that a diagnosis of renal disease would have been made for those subjects. In an effort to control for the effect of surveillance bias, we created a second model where all persons with chronic diseases were removed. Evidence of an association between renal disease and PPI exposure remained (OR = 2.25) in the sample population without chronic diseases (Table 5).

Appropriate control of confounding variables is always important in the analysis of observational studies. In order to produce groups with similar important baseline characteristics, we matched cases to multiple controls based on age, gender, county of residence, and initial date of coverage. Additionally, we controlled for other causes of renal disease and health status, both directly and with propensity scores. It is possible that even after controlling for these known confounders, some residual confounding variables remain. It is also possible that additional confounding variables exist, such as OTC NSAID use, which could not be controlled for in the model, which is a limitation inherent to all observational studies.

## Conclusions

The results of this nested case-controlled study affirm an association between renal disease and PPI use. Our study revealed that patients with a renal disease diagnosis were twice as likely to have a previous prescription for a PPI. Therefore, it is necessary that pharmacist and physician awareness and recognition of patient complaints or clinical manifestations of this potentially harmful event are increased. Furthermore, it is important that future research seek to establish a definitive causal relationship.

## Abbreviations

AIDS: (Acquired immune deficiency syndrome); AIN: (Acute interstitial nephritis); CI: (Confidence intervals); H2 Blocker: (Histamine-2 receptor antagonists); ICD-9: (International classification of diseases, 9th revision); NSAID: (Non-steroidal anti-inflammatory drugs); OR: (Odds ratios); OTC: (Over-the-counter); PPI: (Proton pump inhibitors).

## Competing interests

The authors declare that they have no competing interests.

## Authors’ contributions

DSC conceived the study, participated in the design of the study and helped draft the manuscript. GLC participated in the design of the study, interpretation of the finding and helped draft the manuscript. DGK participated in the conception and design of the study, performed data collection and analysis, and helped draft the manuscript. All authors read and approved the final manuscript.

## Authors’ information

This paper was presented as a poster presentation at the 2006 Annual Meeting of the Americal College of Clinical Pharmacy, St. Louis, MO, October 26–29, 2006. GLC holds a Pharm.D. and Master of Science in Epidemiology and is an Assistant Professor in the Department of Pharmacy Practice at the University of Nebraska Medical Center. DSC is a Pharm.D. with additional credentials as a Board Certified Pharmacotherapy Specialist (BCPS) who is an Assistant Professor in the Department of Pharmacy Practice at the University of Nebraska Medical Center. DGK is a Ph.D. and M.B.A. who is an Assistant Professor in the Department of Pharmacy Practice at the University of Nebraska Medical Center.

## Pre-publication history

The pre-publication history for this paper can be accessed here:

http://www.biomedcentral.com/1471-2369/14/150/prepub
